# Muscle Synergies Control during Hand-Reaching Tasks in Multiple Directions Post-stroke

**DOI:** 10.3389/fncom.2018.00010

**Published:** 2018-02-23

**Authors:** Sharon Israely, Gerry Leisman, Chay C. Machluf, Eli Carmeli

**Affiliations:** ^1^Department of Physical Therapy, University of Haifa, Haifa, Israel; ^2^National Institute for Brain & Rehabilitation Sciences-Israel, Nazareth, Israel; ^3^Signal and Image Laboratory, Faculty of Electrical Engineering, Technion - Israel Institute of Technology, Haifa, Israel

**Keywords:** muscle synergies, post stroke, motor control, variance accounted for (VAF), electromyography (EMG), hand reaching, non-negative matrix factorization (NMF), modulation

## Abstract

**Purpose:** A muscle synergies model was suggested to represent a simplifying motor control mechanism by the brainstem and spinal cord. The aim of the study was to investigate the feasibility of such control mechanisms in the rehabilitation of post-stroke individuals during the execution of hand-reaching movements in multiple directions, compared to non-stroke individuals.

**Methods:** Twelve non-stroke and 13 post-stroke individuals participated in the study. Muscle synergies were extracted from EMG data that was recorded during hand reaching tasks, using the NMF algorithm. The optimal number of synergies was evaluated in both groups using the Variance Accounted For (VAF) and the Mean Squared Error (MSE). A cross validation procedure was carried out to define a representative set of synergies. The similarity index and the K-means algorithm were applied to validate the existence of such a set of synergies, but also to compare the modulation properties of synergies for different movement directions between groups. The similarity index and hierarchical cluster analysis were also applied to compare between group synergies.

**Results:** Four synergies were chosen to optimally capture the variances in the EMG data, with mean VAF of 0.917 ± 0.034 and 0.883 ± 0.046 of the data variances, with respective MSE of 0.007 and 0.016, in the control and study groups, respectively. The representative set of synergies was set to be extracted from movement to the center of the reaching space. Two synergies had different muscle activation balance between groups. Seven and 17 clusters partitioned the muscle synergies of the control and study groups. The control group exhibited a gradual change in the activation in the amplitude in the time domain (modulation) of synergies, as reflected by the similarity index, whereas the study group exhibited consistently significant differences between all movement directions and the representative set of synergies. The study findings support the existence of a representative set of synergies, which are modulated to execute movements in different directions.

**Conclusions**: Post-stroke individuals differently modulate the activation of synergies to different movement directions than do non-stroke individuals. The conclusion was supported by different muscle activation balances, similarity values and different classifications of synergies among groups.

## Introduction

In the upper limb, numerous muscles with thousands of motor units require the control of the motor system even when executing simple daily tasks, such as hand reaching. Direct control on these structures requires simultaneous control over large degrees of freedom, which in turn, imposes a significant computational burden on the Central Nervous System (CNS). Given the nature of controlling such a complex system, an alternative mechanism for motor control was suggested, in which the brain's cortex controls and modulates a simple combinatorial system to control the vast movement repertoire (Bizzi and Ajemian, 2015). In the context of this paper, modulation of synergies will be referred to changes in the amplitude of activations of synergies in the time-domain. This combinatorial system is a composite of discrete numbers of building blocks, i.e., muscle synergies within the brainstem and spinal cord. Each synergy activates a fixed action pattern of muscles expressing a sub-movement or reflexive behavior (Levine et al., 2014). During execution of compound movement the CNS flexibly combines these synergies, and modulates their amplitude and timing of activation (Tresch and Jarc, 2009).

A common method for investigating the properties of synergies, decomposes EMG signals to discrete number synergies, using Non-negative Matrix Factorization (NMF) (Tresch et al., 2006). Comprehensive understanding of the control mechanisms post-stroke, may promote better understanding of the underlying mechanisms of the accompanied motor impairments. From a computational perspective, this model simplifies the movement control mechanisms in a way that places attractive challenges to uncover its anatomical and physiological properties in order to enhance motor performance (Muceli et al., 2010).

Brain lesions affect motor performance in various ways, depending on the location and extent of the damaged area, and the integrity of the corticospinal tract (CST) (Seitz and Donnan, 2015; Puig et al., 2017). Despite a multitude of therapeutic interventions available for post-stroke individuals, considerable numbers of patients still sustain major motor impairments, independent of the rehabilitation paradigm or regime implemented.

The CST is considered to play a crucial role in regulating the upper extremity in humans especially for hand and finger control. While monosynaptic connections in the ventral horn are believed to underlie dexterity skills in primates, the majority of CST terminals are connected via interneurons (Riddle and Baker, 2010). The reticulospinal tract (RST), on the other hand, was previously though to control postural movements. Recent findings, however, suggested that it also has projections to proximal muscles, forearm muscles, and also monosynaptic connections to intrinsic hand muscles (Davidson and Buford, 2006; Riddle et al., 2009; Riddle and Baker, 2010). While both pathways converge on similar interneurons, other interneurons have either inputs from the CST or the RST. While the CST has synapses at both the intermediate and the ventral zones, the RST terminates at the intermediate zone. Accordingly, as opposed to the common notion, it seems that the RST, has also considerable impact on distal control.

In the context of motor control post-stroke, impaired downstream messages transmitted by the CST might be partially compensated by other RST neurons and rubrospinal tracts to control motor outputs (Seitz and Donnan, 2015; Owen et al., 2017; Puig et al., 2017). This, in turn, may shift, in some ways, the target interneurons in the spinal cord that receive these messages and assumingly effect the activation of muscle synergies. This might be manifested as altered activation of preserved muscle synergies, or as modifications in the muscle activation balances within a synergy. Nevertheless, current data using machine learning algorithms to extract muscle synergies from EMG data show equivocal findings. Therefore, currently there is no good evidence of how it is that cortical stroke affects muscle synergies (Cheung et al., 2009; Roh et al., 2013, 2015).

Previous studies investigated the impact of cerebral stroke on the synchronization of synergies and the muscles activation balances within synergies, i.e., the inner structure of synergies (Cheung et al., 2009; Roh et al., 2013, 2015). With regard to mildly impaired patients, Cheung and colleagues observed preservation of the structure of synergies, and therefore stated that, apparently, the changes in the EMG muscle patterns reflect alteration in the modulation of synergies by higher brain centers (Cheung et al., 2012). Roh and collaborators, on the other hand, observed alterations in the structure of synergies in mildly impaired patients (Roh et al., 2015), stating that the reduction in corticospinal inputs may shift the encoding site to lower levels such as the brainstem and spinal cord (Roh et al., 2013).

In more impaired post-stroke individuals, it was suggested that there is a change in the internal structure of synergies (Cheung et al., 2012; García-Cossio et al., 2014; Roh et al., 2015). Some of these studies reported that the impaired synergies reflect mergence of the healthy synergies (Clark et al., 2010; Cheung et al., 2012; García-Cossio et al., 2014). Garcia-Cossio and colleagues reported that subcortical lesions had negative impact on the number of shared synergies, between the affected and less affected extremities (García-Cossio et al., 2014). It was additionally suggested that fewer synergies were extracted in chronic post-stroke patients with higher spasticity, expressing the impaired ability to isolate the movement of limb-segments (García-Cossio et al., 2014).

Investigating the modulation of synergies during hand reaching in post-stroke individuals is complex, due to the inherent motor impairments of patients. A recent study evaluated the changes in task-specific muscle synergies post-stroke during the execution of supported hand reaching task in two dimensions. The study results indicated a greater similarity of non-stroke synergies than post-stroke synergies compared to a baseline set of synergies (Li et al., 2017). However, the task constraints make it difficult to deduce the alteration in the control mechanisms between healthy and post-stroke individuals, given the paradigm in which synergies should be reflected independent of task-constrains (Cheung et al., 2012; d'Avella and Lacquaniti, 2013). Others have studied the direction modulation of muscle synergies post-stroke during isometric force production in mildly to severely impaired patients (Roh et al., 2013, 2015). Therefore, there is scarce evidence regarding the way individuals post-stroke modulate the activation of synergies in hand reaching for different movement directions.

Two studies with non-stroke participants reported that hand-reaching for different directions might be successfully represented by scaling a small number of muscle synergies (Muceli et al., 2010; d'Avella and Lacquaniti, 2013). Among these two studies, Muceli et al. (2010) used cross-validation techniques to investigate how synergies from a certain movement direction may describe movements to other directions (Muceli et al., 2010). The authors reported that extracting synergies from a combination of three targets allowed good reconstruction of the EMG, recorded from movements that were executed to other directions. Semprini et al. (2016) applied the space-by-time NMF algorithm to decompose wrist movements in three dimensions, under four different force conditions (Semprini et al., 2016). The study results indicated that the similarity between the synergies of each of the participants were correlated with the average set of synergies, which in turn was validated as a representative set of synergies. The authors suggested that high to moderate correlation coefficient values allowed the average set of synergies to be set as a representative set of synergies.

In this study we aimed to compare the modulation properties of muscle synergies in post-stroke individuals, compared to non-stroke individuals during hand reaching tasks in multiple directions. This might shed light on the impact cortical stroke has on spinal control mechanisms, and perhaps indicate potential inherent shifting in the efferent pathways. We hypothesize that the EMG data of patients after a stroke might accurately be captured by discrete numbers of muscle synergies with different modulation properties induced by impaired muscle recruitment.

## Methods

### Participants

Twelve healthy volunteers (control group) and 13 post-stroke individuals (study group) participated in the study. The study group participants included seven males and six females, who were 21.77 ± 11.938 days post-stroke, demonstrating mild motor impairments, as indicated by mean Fugl-Meyer scores of 50.769 ± 7.037. The study was granted research ethical approval by both the University of Haifa (ID number 273/16), and by the Bait-Balev Rehabilitation Center Institutional Review Boards (ID number BB0006/16). The study was performed in accordance with the Declaration of Helsinki. All subjects signed an informed consent form.

### Equipment

**Figure 1:**
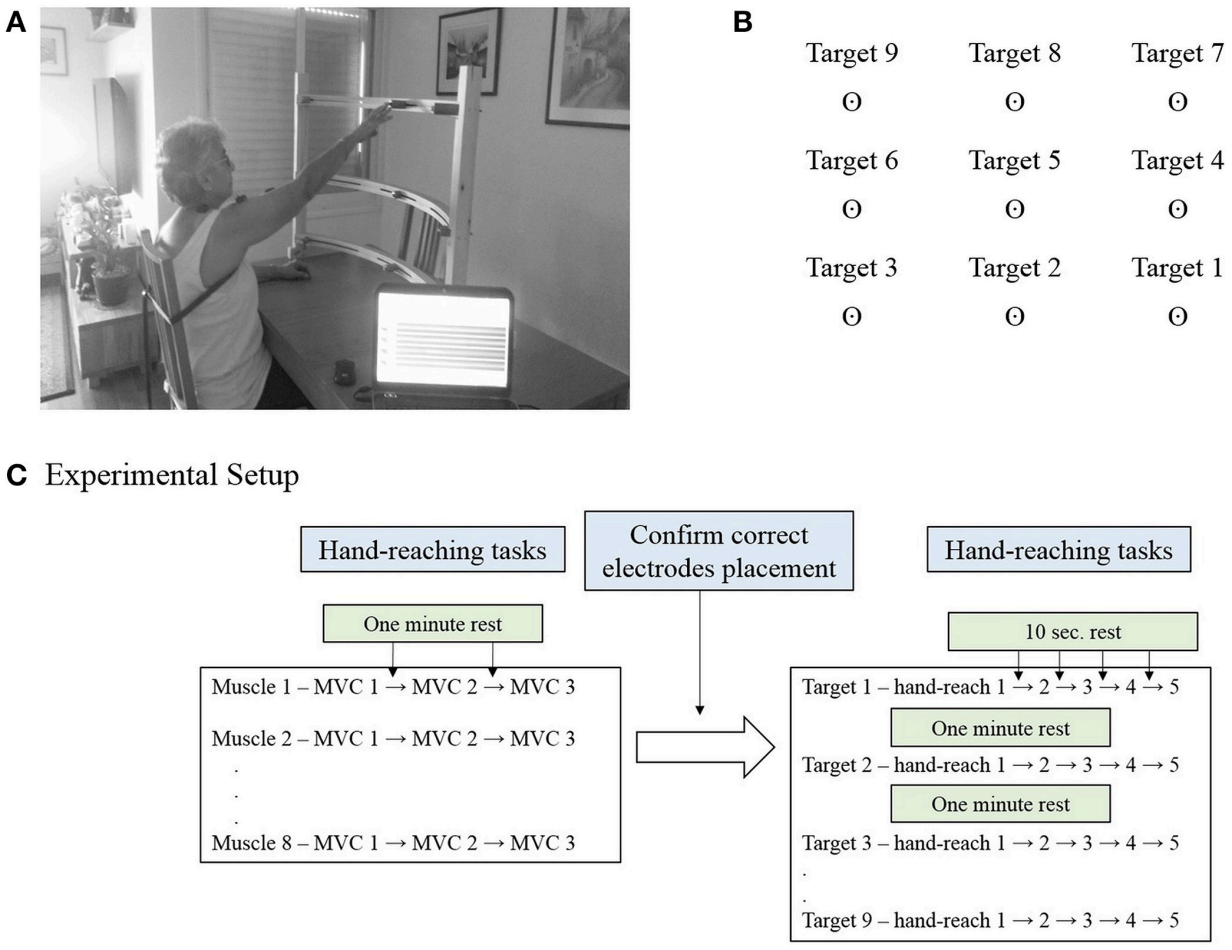
**(A)** The Hand-Reaching Spatial Device. Participants were asked to reach with their dominant hand (control group) or the more impaired hand (study group) to nine different targets that were located in each participant's maximum hand-reaching range of motion. The photographed participant signed an informed consent form to permit publication of this photo. **(B)** Representation of the order and direction of the targets for a person with dominant right hand. **(C)** Experimental setup. Participants were executed three maximal isometric contractions (MVC) for each muscle, with 1 min rest between tests. During these tests the experimenter confirmed correct placement of the electrodes. This was followed by five hand-reaching movements for each of the nine targets, in a fixed order. Participants were aware of the target direction before the execution of the reaching task.

The Hand-Reaching Spatial Device (Figure 1A) is an adjustable, simple tool allowing standardization of hand pointing movements for nine different directions between different participants. It is composed of two vertical rods to which are attached three semi-circular shelves. Each shelf contains three movable pointing pins that can be adjusted left and rightward to accommodate the variable arm length of each participant. The lowest shelf was located 10 cm above the table; the middle was located 35 cm above the table and highest 55 cm above the table.

For each participant the Hand-Reaching Spatial Device was located at the maximum hand reach distance in front of the tested shoulder. The side pins were located at a 45° angle to the shoulder joint to both sides. The arrangement of the targets on the Hand-Reaching Spatial Device was designed to cover the majority of hand-reaching movements.

### Electromyography

Surface EMGs were recorded (Trigno 8, Delsys, Boston, MA) from eight muscles of the shoulder girdle and arm: trapezius; deltoid anterior, medial, and posterior fibers; and pectoralis major; infraspinatus; biceps; triceps. Electrodes were placed in accordance with the guidelines of the Surface Electromyography for the Non-Invasive Assessment of Muscles–European Community Project (SENIAM) (Hermens et al., 1999). Maximum voluntary contractions (MVCs) were performed, while muscle activity was monitored by the EMG device. This procedure verified correct electrode placement and normalization. MVC tests were performed for all muscles according to manual muscle testing techniques (Hislop et al., 2002). Participants were asked to perform a full range of motion involving the activation of the tested muscle against gravity with no additional resistance to movement. If a full active range of motion was obtained, participants were asked to locate the extremity in a predefined posture, in which the tester applied gradually increasing resistance until muscle failure. In cases where participants were not able to complete a full range of motion against gravity, the test was repeated in an alternative posture to eliminate the effects of gravity (Hislop et al., 2002). One-minute rest periods followed each MVC to limit the possibility of fatigue. EMG signals were band-pass filtered (20–450 Hz), and sampled at 2000 Hz.

### Protocol

The MVC was measured by standard muscle testing (Hislop et al., 2002). Participants sat in front of a table with the forearm resting in a comfortable position. The Hand-Reaching Spatial Device was located as mentioned above. Participants were requested to point to each target five times according to voice prompting that was activated by the EMG software every 10 s, for 45 pointing movements. One to 2 min rest was allowed between reaching to different targets. The order of pointing targets was constant for all the participants, and explained for the participants before the procedure. Figure 1B illustrates the order of the targets for a person with right hand dominance. Accordingly, participants carried out five hand-reaching movements to target 1, followed by five hand-reaching movements to target 2, then target 3 and so on. The order of targets for a left hand dominant person was horizontally mirrored, but fixed in the vertical dimension such that target 1 was on the left- down and target 9 was on the right-up. Figure 1C details the experimental setup for a single participant.

### Data analysis

#### EMG preprocessing

Data analysis was performed using Matlab (The MathWorks, Inc.). EMGs were demeaned, followed by an RMS calculation using an overlapping window of 50 samples (25 ms around each time point). Mean baseline EMGs for each trial was subtracted from the averaged data for the sequence of reaching movements. Hence, the EMG data for each trial, a vector whose dimension was 8 (the number of muscles recorded), corresponded to active force generation beyond any residual baseline muscle activity. The power of the EMG for each muscle was normalized in accordance to the corresponding MVC of the same muscle.

#### Identification of muscle synergies

The NMF algorithm originally used by Lee and Seung was applied to identify muscle synergies and their activation weights (Lee and Seung, 1999, 2001). An EMG pattern recorded in hand-reaching movements was modeled as a linear combination of a set of *N* muscle synergies, each of which specified the relative level of activation across eight muscles, and activated by a time-varying activation coefficient (Tresch et al., 2006; Roh et al., 2012):

(1) $$\begin{array}{l} V^{N\times M}\approx W^{N\times R}\cdot H^{R\times M} \end{array}$$

Where *V* is the EMG data set matrix with *N* as the number of muscles (8 muscles), *M* as the number of time samples, *W* is the synergy matrix and *H* is the coefficient matrix. *W* is *N*×*R* is a matrix with *R* synergies, *N* is the number of muscles, and *H* is an *R*×M matrix with *R* synergies and *M* is the number of time-samples. Thus, each column of *W* represents the weights of each muscle for a single synergy, and each row of *H* represents how much the corresponding synergy was activated or used to generate force. In this model, it is possible for each muscle to belong to more than one synergy, and thus the EMG of any single muscle might be attributed to simultaneous or sequential activations of several muscle synergies. The NMF reconstruction accuracy was measured by evaluating the amount of data variation explained by the model, i.e., Variance-Accounted-For (VAF) that will be further detailed.

#### Defining the optimal number of synergies

Two criteria were applied to determine the optimal number of synergies: (1) mean squared errors (MSE); (Cheung et al., 2005); and (2) the Variance Accounted For (VAF) (Cheung et al., 2005; Roh et al., 2013). The optimal number of synergies was identified by the number of muscle synergies at which the VAF curve changed sharply according to the MSE value (Cheung et al., 2005; Roh et al., 2015). The first method suggested fitting portions of the VAF curve to a straight line using the least squares technique. Initially all data points on the VAF curve were included, and then the 2nd to 7th points and so on until only the 5th and 7th points were included. The correct number of synergies could then be estimated, as the first point on the VAF curve at which the linear fit of all points from that point to the 7th point produced a small MSE. Using the second method, the optimal number of synergies was defined as the minimum number of synergies that achieved a mean VAF > 85%, with less than a 6% increase in mean VAF upon addition of another synergy (Roh et al., 2013).

The NMF algorithm requires the number of synergies extracted to be specified before the application of the algorithm. Therefore, for each data set, the VAF was calculated while changing the number of synergies from 1 to 7. The VAF values were calculated using the equation according to d'Avella et al. (2006) as follows:

(2) $$\begin{array}{l} VAF\left(H\right)=100\%\times \left(1-\frac{||V-WH|{|}_{2}^{2}}{||V-\bar{V}|{|}_{2}^{2}}\right) \end{array}$$

**Figure 2:**
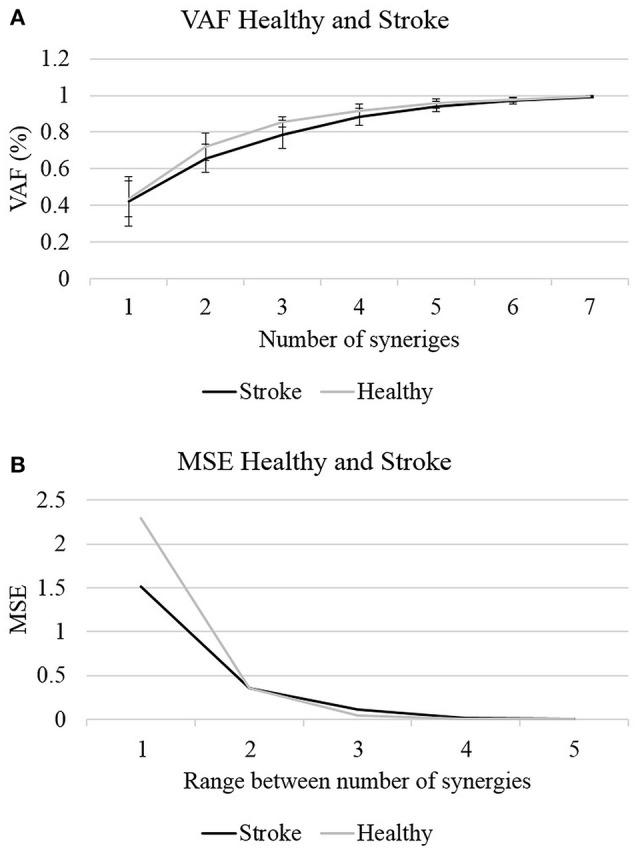
The optimal number of synergies was determined according to two criterions: the VAF **(A)** and MSE **(B)**. Four synergies could explain >85% of the data variances with MSE value <0.02 in both groups, to be set as the optimal number of synergies.

Where *V* is the original matrix, and *W* and *H* are the derived factorized matrices. In the denominator the mean vector *V* of each row in *V* were subtracted from each data point in each row in *V*. Figure 2 illustrates the VAF and VAF variances against the number of synergies for each group.

#### Defining representative set of synergies of non-stroke individuals

**Figure 3:**
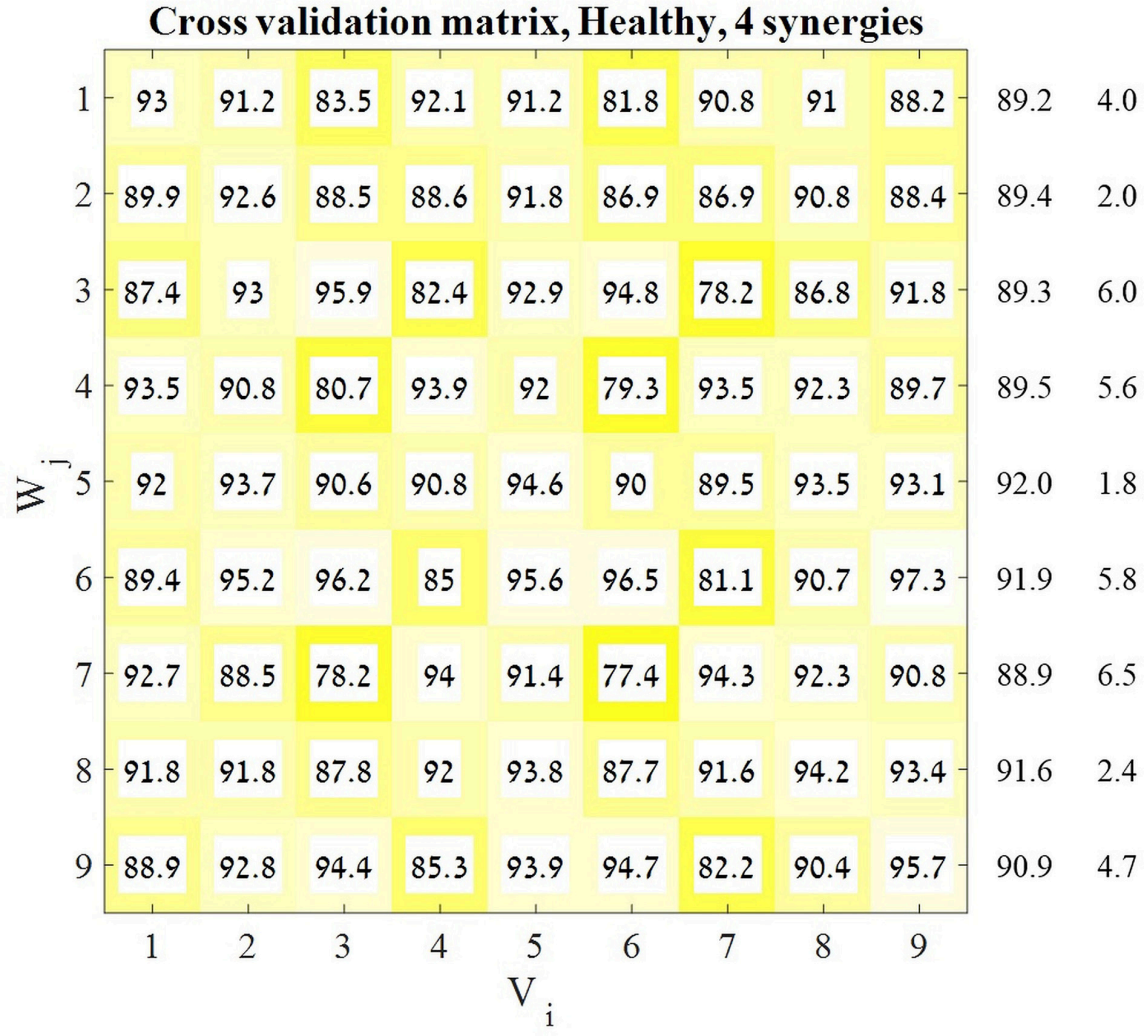
Cross-validation matrix. Each *V*_*i*_ matrix was decomposed by each *W*_*j*_ matrix. Each cell in the cross-validation matrix represents the corresponding VAF for each calculation. The representative set of muscle synergies was determined according to the highest mean VAF across the rows of the matrix. The values on the right of the matrix are the means and SD's of each corresponding row. In order to validate this decision two additional indices were computed.

Our aim in this stage of analysis was to establish whether a set of synergies exists that controls any reaching movement in space. Therefore, we investigated how movement in certain directions could account for movements in other directions. We pooled the EMG data for each movement direction separately across the eight muscles and concatenated it for the whole sample. In that way the derived set of synergies would have to account for the variances between different subjects, but would also be specific for that direction alone. We applied the NMF separately for each movement direction according to the equation:

(3) $$\begin{array}{l} V_{i}\approx W_{i}\cdot h_{i} \end{array}$$

where *i* is the target number, which corresponded to specific movement direction in space. In this stage of the analysis *V*_*i*_ (the EMG matrix) was given as an input for each target, *i* ∈ [1, 9], and matrices *W*_*i*_, and *h*_*i*_ were updated iteratively. The study procedure included reaching for nine different target directions in space, allowing us to further investigate if there was a single set of synergies that could account for movements in other directions. This was done by using a cross-validation technique between the *V*_*i*_ matrices and the *W*_*j*_ matrices by applying a modified version of the NMF algorithm. This was followed by corresponding VAF calculation changing the number of synergies (*d*) from only 3 to 5, and not from 1 to 7 based on the results of the NMF for all the participants and for all targets, as detailed in the results section. In the modified version of the algorithm, both *V*_*i*_ and *W*_*j*_ (the synergies matrix) were given as an input. Only the coefficients matrix was updated and outputted. For each pair of a target matrix and synergies matrix (*V*_*i*_ and *W*_*j*_), a distinct coefficient matrix was generated, which we will refer to as *H*_*ij*_. The dimensions of *h*_*i*_ in Equation (3) were calculated according to Equation (1), with R rows as the number of synergies and M columns as the number of samples in *V*_*i*_. The dimensions of *h*_*ij*_, on the other hand were *R* rows (as in *h*_*i*_), but with different number of columns (*M*) as the number of samples in the *V* matrices were different for different target directions. The cross-validation process of the modified NMF was carried out for each combination of a data matrix *V*_*i*_ (of target *i*) and a synergy matrix *W*_*j*_ (of target *j*), resulting in 9 × 9 matrices *H*_*ij*_ including *V*_*i*_ and *W*_*i*_, (i.e., overall, 81 distinct *H*_*ij*_ matrices resulted from the cross-validation process). Each *H*_*ij*_ was *R*×*M* dimension matrix, in which *R* was fixed according to the number of synergies, but with different number of samples *M*. For every *i, j* ∈ [1, 9], we factorize *V*_*i*_ such that *W*_*j*_*H*_*ji*_ ≈ *V*_*i*_. The representative set of muscle synergies was chosen by calculating the VAF for each of the 9 × 9 factorizations:

(4) $$\begin{array}{l} VAF\left(H_{ij}\right)=100\%\times \left(1-\frac{||V_{i}-W_{j}H_{ij}|{|}_{2}^{2}}{||V_{i}-\bar{V_{i}}|{|}_{2}^{2}}\right) \end{array}$$

assuming that consistent high values of *VAF*(*H*_*ij*_) for a specific *V*_*i*_ may indicate that the synergies obtained from movements in this direction may accurately explain movement in other directions. Thus, for each predefined number of synergies we received a 9 × 9 *matrix* (Figure 3) in which each cell represented the accountability of a given synergy (row) to a specified direction (column). Each row in the resulting matrix represented the overall “performance” of the appropriate set of synergies, and so the row with the highest average VAF was chosen to be the representative set of synergies for the next stages of analysis.

#### Comparing between synergies of different humans and between different groups

**Figure 4:**
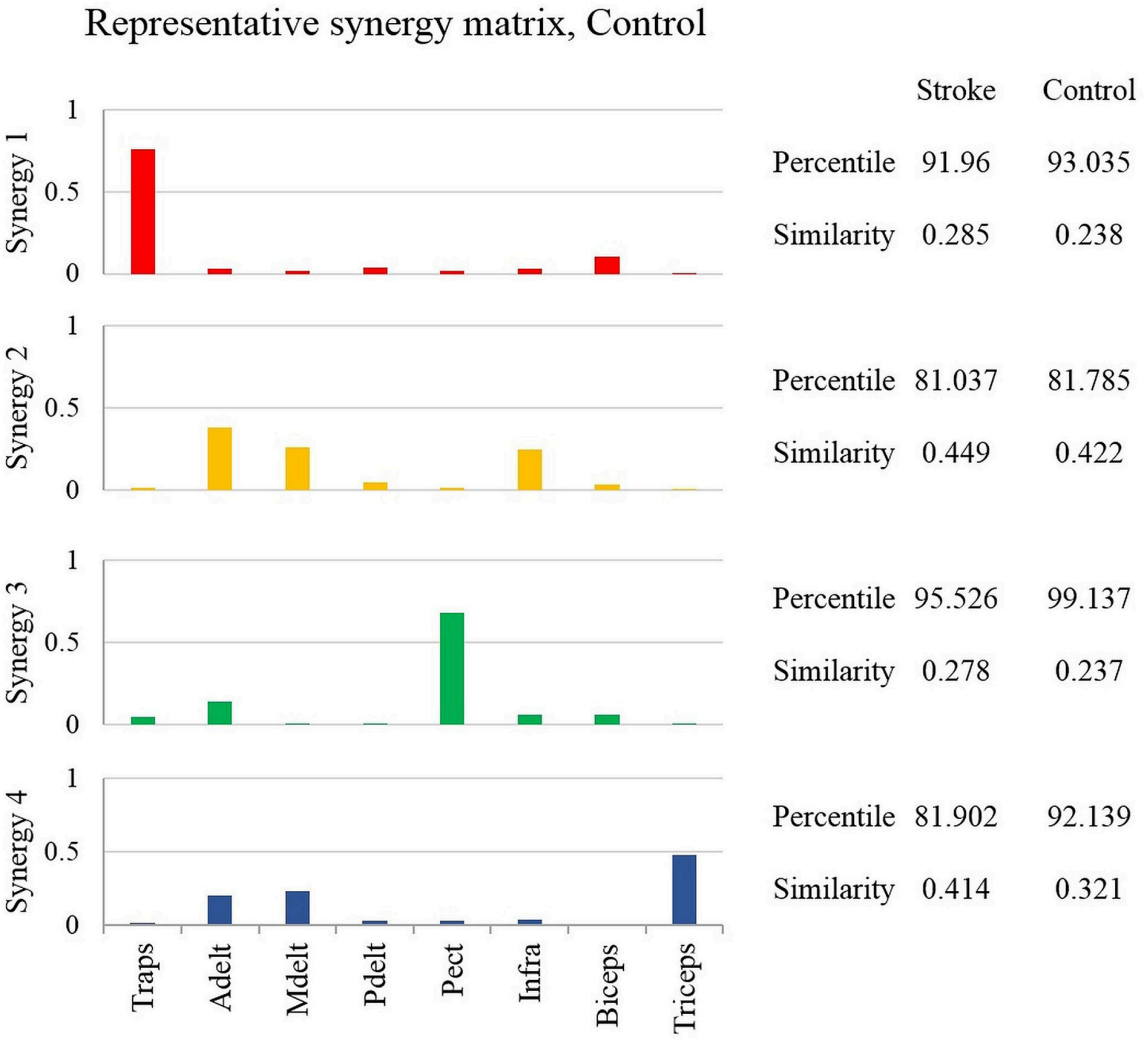
The representative synergy matrix *W*_5_. Each synergy is composed of fixed pattern of muscle activity. The height of the bars refers to the amplitude of activation of a single muscle relative to the other muscles. Since all the muscles that were monitored by the EMG device, composed entire synergy, the amplitude of each synergy sums to one. The similarity between the synergies of each of the participants from both groups and the representative set of synergies was calculated and averaged, and written on the right side of the plot. The similarity index receives values from zero (high similarity) to one (completely different matrices). Determining the identity of synergies to the representative set of synergies, based on the percentile of similarity between the tested synergies among 10^6^ combinations of these synergies, randomly ordered. High percentile values suggest that the tested synergies are similar relative to the same synergies, randomly ordered.

**Figure 5:**
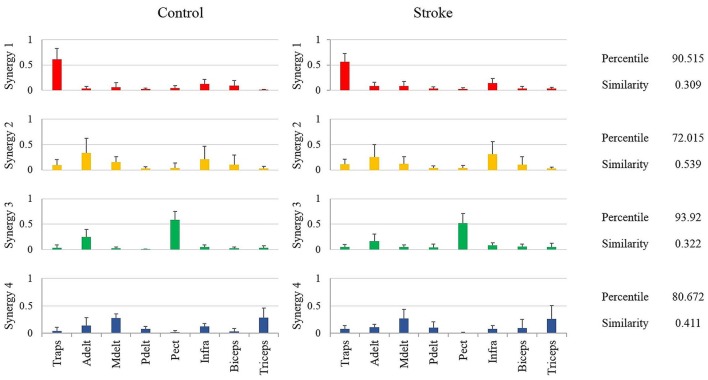
Mean synergies of groups, their similarity index and mean percentile of similarity. The mean percentile of similarity was calculated compared to 10^6^ pairs of randomly ordered synergies. The similarity and percentile of similarity were calculated between each participant from the control group and each participant from the study group.

This stage of analysis was carried out based on the studies by Cheung et al. (2005) and Roh et al. (2012). Two statistical methods were applied to evaluate the similarity between group synergies. In both methods, synergies of each participant were extracted from the pooled EMG data from all target directions. In the first method, the similarity and the percentile of similarity between the synergies of each participant and the representative set of synergies was computed, and averaged for each group (Figure 4 on the right side of the plot). Using the same technique in the second method, each synergy from each participant from the control group was compared to the synergies of all the participants from the study group (Figure 5 on the right side of the plot).

In both methods the percentile of similarity and the similarity between synergies were calculated as follows (Datasheet 1, Supplementary Material): Each of the tested synergies was matched with the corresponding set of synergies, and accordingly ordered to provide the lowest Euclidian distance. Since eight muscles were monitored, each synergy could be randomly ordered in 8! = 40320 possibilities. To evaluate the chance level of similarity, we first generated 1,000 random synergies for each synergy within each set of four synergies to be compared. Each of the 1,000 random sets of synergies consisted of the muscle weights from the evaluated synergy, but randomly shuffled in order.

We then calculated the similarity between all possible pairs of random synergies from the two tested synergies (1,000 × 1,000 = 10^6^ pairs in total), and compared it to the similarity between the non-shuffled sets of synergies. Each two sets of synergies were indicated to be similar if their mean similarity index rose above the 90th percentile of the distribution of the random similarity indices. Comparisons between groups, in the first method, were applied by an independent *t*-test, using the mean percentile of similarity index and the mean similarity values between groups.

The similarity index was calculated based Euclidian distance between each corresponding synergies and divided by two to be normalized to one as follows:

(5) $$\begin{array}{l} Similarity\;Index\left(rep,W\right)=\;\frac{\sum_{i=1}^{8}|W_{i}^{rep}-W_{i}^{ind}|}{2} \end{array}$$

Where *R* ∈ [1, 8] was the muscle number, and *ind* ∈ [1, 12] in the control group or *ind* ∈ [1, 13] in the stroke group was the number of participant, *rep* stands for representative synergies. Each *W*_*i*_ matrix was *i* × *R* matrix, with *R* synergies. Since the sum of each column in matrix *W* is one, so the maximal score for similarity between each two matrices was one.

#### Comparing between groups synergies by cluster analysis

Hierarchical cluster analysis was applied for each group separately. For each participant, synergies were extracted from the pooled EMG data set, and therefore account for all movement directions. Each of the four synergies of each participant, were ordered in a 1 × 8 vector. The synergies of the whole groups were ordered in a 48 × 8 matrix in the control group or a 52 × 8 in the study group. The optimal number of clusters was determined as the minimum number of clusters allowing portioning the data such that there was not more than one synergy in each cluster from a given subject (Cheung et al., 2005; d'Avella et al., 2006; Roh et al., 2015). Intuitively, it means that synergies of a single participant differs in their muscle composition, and on the other hand, corresponding synergies of different participants are similar in their muscle composition, and therefore could be classified to be in the same cluster.

**Figure 6:**
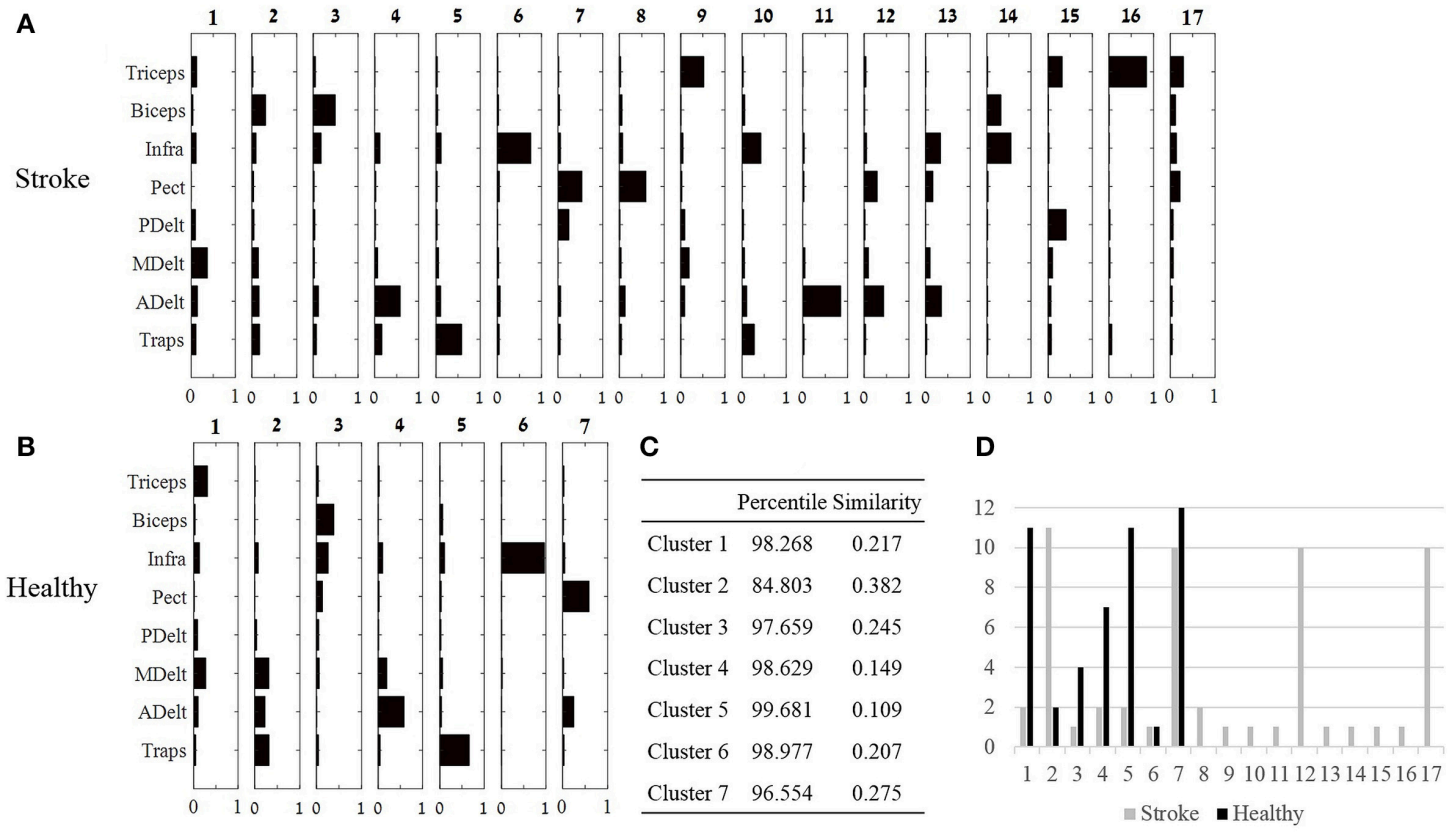
Cluster analysis. **(A,B)**: Seventeen and seven clusters optimally represent the synergies of the study and control group, respectively. Large number of clusters post-stroke suggests that individuals recruit different synergies, whereas decreased number of clusters in the control group, suggest that different individuals uses similar synergies. **(C)** The optimal similarities of seven clusters between groups and percentile of similarity among random synergies were computed. Six out of seven clusters were similar between groups. **(D)** The number synergies (y axis) that were classified to each cluster in both groups.

The similarity between each pair of clusters from both groups was calculated using Equation (5), and illustrated in Figure 6C. Each cluster from the control group was paired with the single closest cluster from the study group, according to the highest similarity between clusters. If two clusters from the control group, matched with the same cluster from the study group, than the pair with the lower similarity value was set as a pair. This process was repeated until all healthy participant clusters were paired with a single cluster from the study group. The results of the cluster analysis including the similarity values between pairs of clusters are plotted in Figure 6. Since each participant has four synergies, under the portioning condition, if four clusters had optimally represented the data that would suggest that all participants shared common synergies. Correspondingly, many clusters for a given group of participants means that participants have different synergies.

#### Methods for validating the representative set of muscle synergies

**Figure 7:**
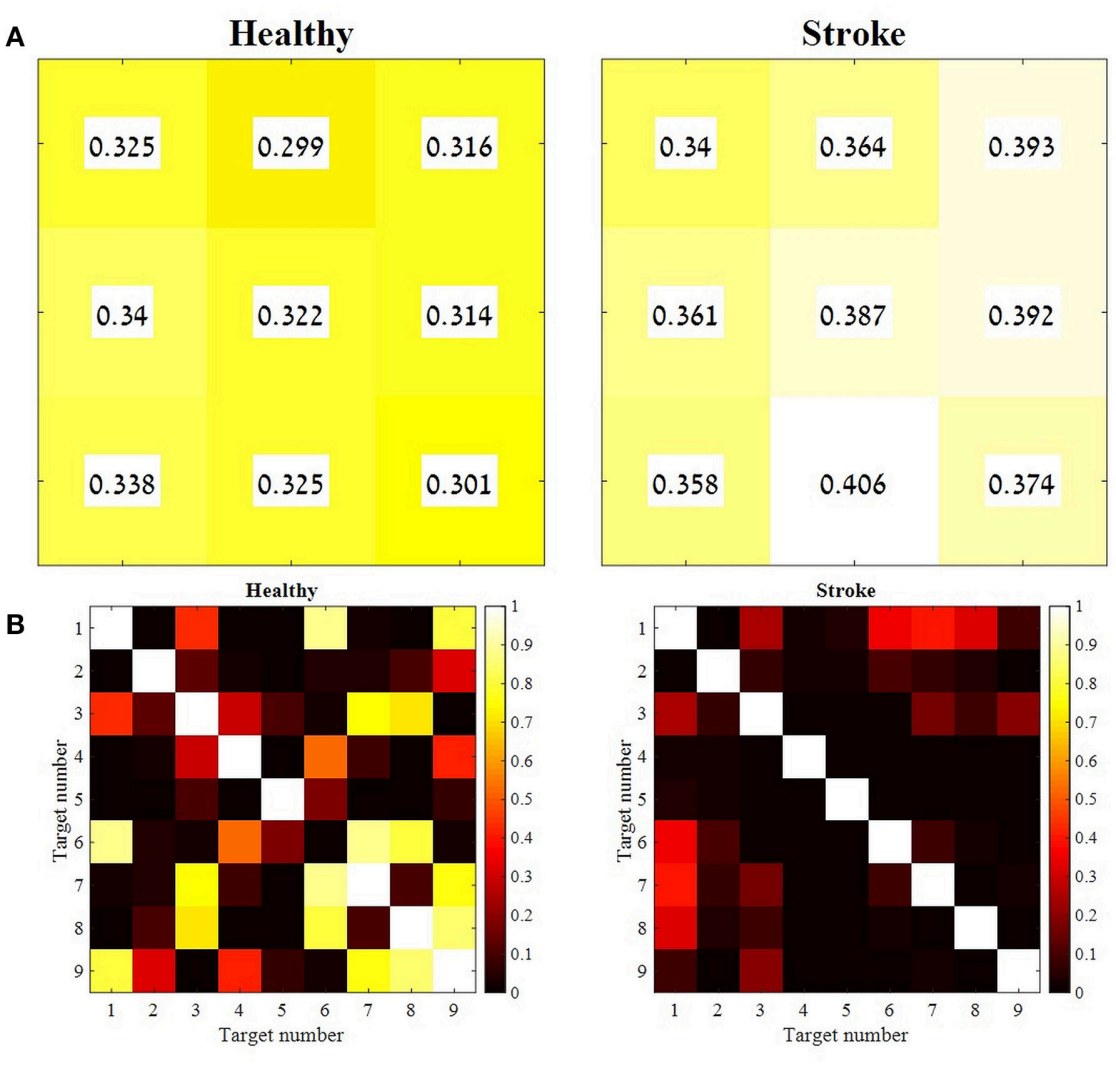
The mean similarity between the representative set of synergies and synergies from all other movement directions, and the level of significance for both groups. For each participant from both groups, synergies were extracted for each movement direction separately. Therefore, each participant represented by nine synergy matrices. The distances between each of the nine synergy matrices and the representative set of synergies were computed and averaged for each group. Each cell in the matrices in **(A)** correspond to a target as indicated in Figure 1B. A paired sample *t*-test was applied to test the differences between the similarity values of each combination of the nine target directions, for group separately **(B)**. The mean similarity values in **(A)** were aligned according to Figure 1B. The similarity index, as indicated in Equation (5) takes values from zero to one, in which zero in complete identity between matrices and one is completely different matrices. Therefore, higher similarity values are indicated by lower values and darker colors, whereas lower similarity values are indicated by higher values and brighter colors. The *p*-values matrices in **(B)** indicate consistent significant differences between different movement directions in the study group (consistent dark colors) compared to variable similarity levels between different movement directions in the control group, which is indicated by different brightness of cell between different movement directions.

In order to validate the choice of the representative set of synergies, two additional statistical methods were applied: (1) the Similarity Index and (2) K-means Cluster Analysis. The Similarity Index was calculated for each participant, between the representative set of muscle synergies and synergies that were extracted from all other movement directions. The similarity values and their similarity percentiles were averaged for each group separately, and plotted in Figure 7 using Equation (5). A weighted correlation matrix (Figure 7B) was calculated using all pairs of target direction similarities. The correlation matrices were averaged for each group separately.

#### Discrimination between different movement directions based on the properties of synergies

The K-means algorithm was applied to study whether the full activation coefficient *(H matrices)* properties of synergies may discriminate between different movement directions. The data used for the K-means were the *H* coefficient matrices of the cross validation procedure. In both groups, each *V* matrix for each direction separately was decomposed by a standard NMF. Then the cross-validation procedure was carried out between each original *V*_*i*_ matrix and the *W*_*j*_ matrices that were extracted from all other directions. For each group the cross validation procedure yielded 81 full coefficient matrices *(H)*. A constant set of 11 features, were extracted from each matrix of each synergy. Each of the *H* matrices features of each synergy was represented as a data point (a single row), which was composed of 44 features (11 *features* × 4 *synergies*). The selected features included six data points equally scattered on the *H* coefficient matrix, time to first peak and its amplitude, time to second peak and its amplitude and the total area under the curve. Peaks were defined using the “MinPeakWidth” function of 500 data points in MATLAB. If either the first or second peaks did not exist, the algorithm substituted the missing data by the mean amplitude and middle time point of the matrix.

**Figure 8:**
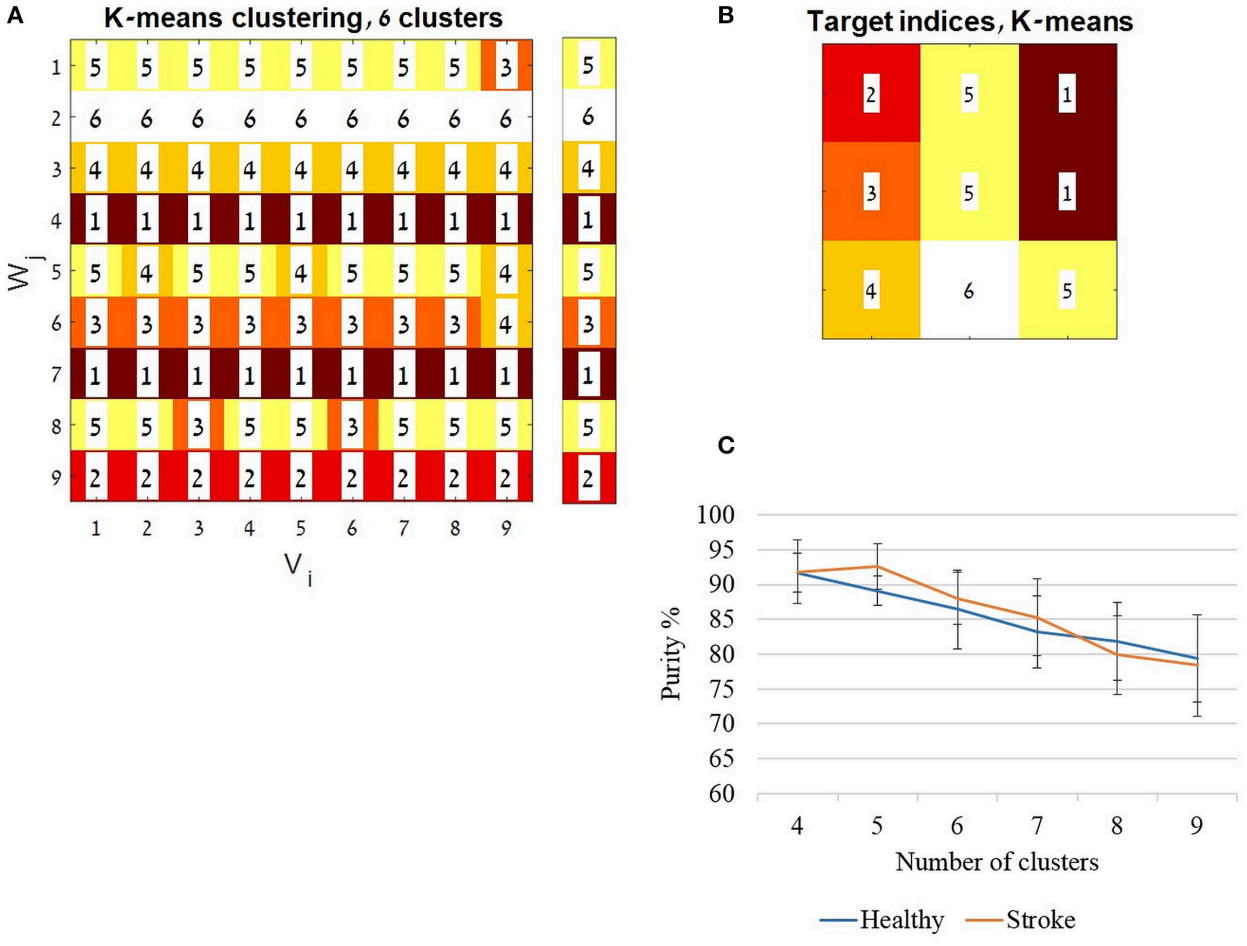
The K-means algorithm. The K-means algorithm was applied for validating the application of a representative set synergies, using the full activation coefficients *(H matrices)*. For each group the cross validation procedure yielded 81 full coefficient matrices *(H)*. A constant set of features (see also method section) was extracted from each matrix. Each of the *H* matrices features was calculated as a data point (a single row), which composed of 44 features (11 *features* × 4 *synergies*). The K-means algorithm was iterated 10 times, changing the number of clusters/ centroids (*K*) from four to nine. The clustering indices were ordered to be aligned with the cross validation matrix (Figure 3) as illustrated in **(A)**. Each row was assigned to a cluster index value given by the MATLAB algorithm, according to the most common index in a row. The assigned indices are shown in the legend bar **(A)**. The accuracy of classification was computed using the purity index (see also method section) for each running of the algorithm with each *K*'s. In **(A)** for example the purity was calculated as follow: ((8 + 9 + 9 + 9 + 6 + 8 + 9 + 7 + 9)/81)^*^100, which resulted in 91.358% accuracy. **(B)** Illustrates the location of the indices ordered according to the target directions as in Figure 1B. **(C)** The average accuracy from the 10 iterations for each *K* was computed and plotted, for each group separately.

The K-means algorithm was iterated 10 times using random centroids and by changing the number of clusters/centroids (*K*) from four to nine. The accuracy of classification was computed using the purity index for each running of the algorithm for each *K*. The purity was defined as the total number of data points that were classified correctly divided by the total number of data points, and multiplied by 100. The correct classification of a cluster was determined according to the most frequent index value in a row of the K-means analysis matrix (Figure 8A). The average accuracy for each *K* was computed and plotted for each group separately (Figure 8C).

#### Direction modulation of activation of synergies in the time domain

**Figure 9:**
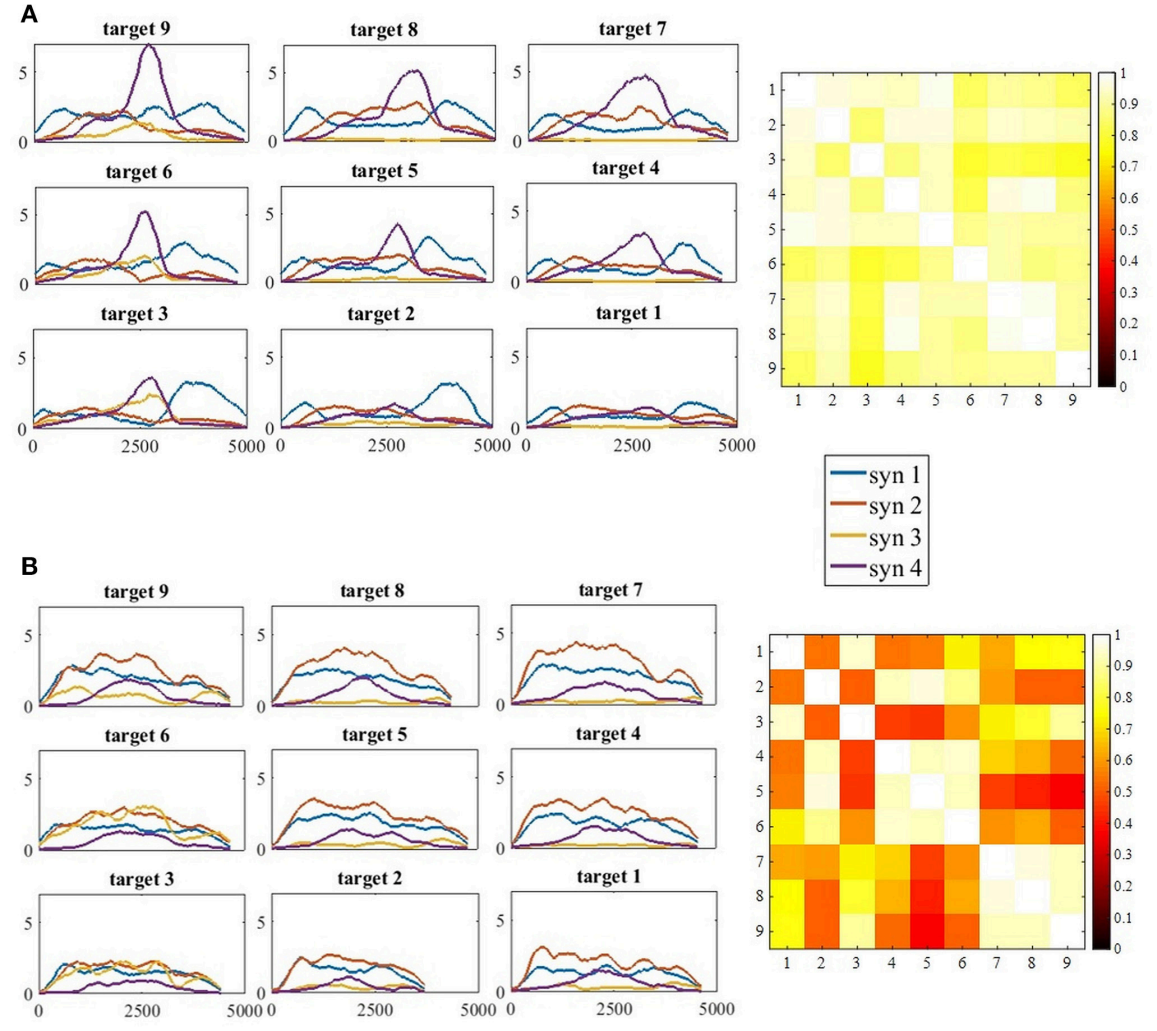
**(A)** The full activation coefficients matrix (H) for each movement direction, of participant 11 from the control group (**A**, Left) and the correlation matrix of the activation coefficient matrices on the right. **(B)** The full activation coefficients matrix (H) for each movement direction, of participant 11 from the study group (**B**, Left) and the correlation matrix of the activation coefficient matrices on the right. The four synergies demonstrated different time- patterns of activation for each of the targets. Synergies were differently regulated by the time-coefficients for different targets. In each subplot on the left side **(A,B)** the x-axis refer to time and the y-axis to the activation coefficient value. Therefore, the values of the y-axis were not normalized. The correlation matrices illustrate high correlations in the control group between all pairs of movement directions, in contrast to the study group that illustrate high correlation values between targets at the same height (4, 5, 6 and 7, 8, 9), but not between different heights (5 vs. 7, 8, and 9).

The functional role of each of the extracted synergies was investigated by plotting the full activation coefficient matrices (*H*) (Figure 9). EMG data matrices of each participant and for each movement direction were decomposed by the representative set of synergies. Accordingly, these temporal activation properties helped to distinguish between different movement- directions. The extracted features that were used to apply the K-means were also used to calculate the correlation matrices (Figure 9 on the right). In each group, nine full activation coefficient matrices (*H*) were factorized by the modified NMF, using *V*_*i*_, *i* ∈ [1, 9] and *W*_5_.

### Statistical analyses

A comparison of the number of synergies between groups as detailed in subsection The Optimal Number of Synergies in Non-stroke and Post-stroke Individuals was carried out using the MANOVA procedure. Between-groups comparisons of the distance of individual synergies from the representative set of synergies (subsection Comparing between Synergies of Different Humans and between Different Groups) were performed using an independent-sample *t*-test. A comparison of the differences between similarity values for different movement directions was carried out for each group separately using a paired-sample *t*-test. A comparison between the similarity values for each movement direction between groups was applied by independent-sample *t*-test. Both procedures are detailed in subsection Validation for the Existence of a Representative Set of Synergies. Correlations of the weighted correlation matrices in subsection Validation for the Existence of a Representative Set of Synergies and Direction Modulation of Activation of Synergies in the Time Domain were Calculated Using the Pearson's Correlation Coefficients. The level of significance was set to <0.05 in all statistical tests.

## Results

### The optimal number of synergies in non-stroke and post-stroke individuals

In the first stage of analysis we aimed to compare the number of synergies which are required to reconstruct the EMG data in both groups, before determining the optimal number of synergies, and further analyzing the properties of these synergies. MANOVA revealed that there were no statistically significant differences in the VAF values between groups, *F*_(7, 17)_ = 1.463, *p* < 0.1; Wilk's Λ = 0.624, partial η^2^ = 0.376. The quality of the NMF to reconstruct the EMG data was evaluated by two measures: MSE and VAF. Figure 2 illustrates the changes in the VAF and MSE values as a function of the number of synergies. Calculating the MSE for the non-stroke and post-stroke groups for three synergies (Figure 2B), yielded MSE values of 0.0409 and 0.108, respectively. Four synergies yielded MSE values of 0.007 and 0.016, and five synergies MSE of 4.7^−4^ and 5.8^−4^. The observed MSE values were significantly higher than the values reported by others (Cheung et al., 2005; Roh et al., 2015), suggesting a decreased similarity of the VAF curve to a straight line.

The application of NMF and a corresponding VAF were conducted separately for each participant. Figure 2 illustrates the two criteria, which were applied to determine the optimal number of synergies: the mean VAF and the MSE. In both the non-stroke and the post-stroke groups, three synergies accounted for 0.856 ± 0.0286 and 0.788 ± 0.075 of the data variances, with respective MSE of 0.04 and 0.108. Four synergies accounted for 0.917 ± 0.034 and 0.883 ± 0.046 of the data variances, with respective MSE of 0.007 and 0.016. Based on Roh's and colleagues method, the optimal number of synergies was defined as the minimum number of synergies that achieved a mean VAF > 85%, with less than a 6% increase in mean VAF upon addition of another synergy (Roh et al., 2013). Four synergies was the minimum number of synergies that met our criteria, and therefore chosen to be utilized for further analyses.

### Defining the representative set of muscle synergies

A previous study has indicated that a representative set of synergies might be modulated to represent different movement direction in space by applying a cross-validation technique (Israely et al., 2017). Here, the same cross-validation method was applied, using a modified version of the NMF algorithm, while presetting the optimal number of synergies to four. This procedure yielded the 9*X*9 cross validation matrix (Figure 3). The representative set of synergies was determined to be the *W*_*j*_ synergy matrix that produces the highest mean VAF values, among all *j* ∈ [1, 9]. Figure 3 demonstrates that the fifth row received the highest mean VAF value of 91.977 ± 1.824 while all the other rows received non-significantly lower mean VAF values. Based on these results *W*_5_ was chosen to be the representative set of synergies.

### The representative set of synergies

Determining the representative set of synergies allowed a simple implementation of comparing synergies between groups. Additionally, a previous report has found that this set of synergies, might be generalized to construct the EMG data from different movement directions (Israely et al., 2017). Figure 4 illustrates the representative set of synergies. The percentile and similarity values on the right side of the plot refer to the mean similarity between individual synergies and the representative set of synergies. The similarity values indicate that three muscles mainly activated synergies two and four. Synergy two was activated by the anterior deltoid, medial deltoid, and the infraspinatus, and synergy four by the anterior deltoid, medial deltoid and the triceps. Synergies one and three, on the other hand were mainly activated by two muscles. Synergy one was activated by the trapezius and biceps, and synergy three by the anterior deltoid and the pectoralis. The anterior deltoid muscle was significantly activated in three synergies. The posterior deltoid was almost not activated as indicated by the representative set of synergies of the healthy participants. The representative set of synergies *W*_5_ (Figure 4) was further used to decompose the EMG datasets of each participant from both groups.

### Comparing between synergies of different humans and between different groups

In method 1, the similarity between the synergies of each participant from both groups and the representative set of synergies was computed by calculating the Euclidian distance between each of the synergies according to Equation (5). An independent sample *t*-test revealed no significant differences between groups. The synergies of study group and the control group participants were similar to the synergies of the representative, with mean similarity percentile of 87.606(7.627) and 91.524(6.943) correspondingly. The mean similarity values were 0.357(0.078) and 0.305(0.088) for the study and the control group correspondingly. According our criteria for similarity, three participants out of 12 from the control group and four out of 13 from the study group demonstrated different synergies from the representative set of synergies.

The representative set of synergies were extracted from the NMF that was applied to target 5, as the cross-validation between *W*_5_ and *V*_*i*_ yielded the highest mean VAF compared to *W*_*j*_ that were extracted from other movement directions. Therefore, the representative set of synergies represents the synergies that were extracted from movements to the center of the hand reaching space. Individual synergies, on the other hand extracted from the pooled EMG data of each participant, including all the nine movement directions. We assume that this non-compatibility influenced the mean percentile of similarity to be dropped below 90 percent in one synergy in the control group and in two synergies in the study group (Figure 4).

Another way of comparing the synergies between groups is by directly analyzing the mean synergies of both groups and then calculating the similarity between them. The bar charts in Figure 5 illustrates the mean synergies of both groups. The mean similarity and percentile of similarity between each pair of corresponding synergies of each pair of participants between groups are illustrated on the right side of the plots. Figure 5 indicates that synergy two and synergy four were not matched between groups according to our similarity criteria. The differences between the muscle compositions of synergies, however, were not significant between groups. Integrating the results illustrated in Figures 4, 5, there is presumably inconsistency in how synergy 2 in both groups had identical low similarity values compared to the representative set of synergies (Figure 4), but group synergies were also distant from each other (Figure 5). A possible explanation for this finding is that synergy 2 from a representative set of synergies expressed the average between synergies 2 of the groups, such that the distance of each group synergies to the representative set was equal in opposite directions.

### Cluster analysis

The third method for comparing between group synergies employed hierarchical cluster analysis. Cluster analysis offers a different perspective for comparing mutual synergies i.e., a cluster, between groups, rather than comparing mean synergies as applied in subsection Comparing between Synergies of Different Humans and between Different Groups. Seven and 17 clusters in the control and the study groups were the lowest number of clusters partitioning the data according to our criteria. The highest similarity between clusters from both groups were computed and illustrated in Figure 6C. Six out of seven of these synergies were similar between groups. The validity of these similarity values should be carefully considered especially for cluster 6, which was found in only one participant in both groups. Other clusters within the seven clusters that were compared were found in at least four participants in at least one group.

Nine clusters in the study group were presented in only one participant (Figure 6D), in contrast to the control group in which a single cluster belonged to one participant. These nine participants' individual clusters were derived from eight participants in the study group, which makes it difficult to distinguish which properties of stroke (i.e., type, location, or extent) might be associated with emergence of “singular clusters.”

The similarity between clusters (Figure 6) and synergies (Figure 5) were also calculated for both groups. In the control group synergy 1 was similar to cluster 5 (similarity of 0.054, percentile of 99.996), synergy 2 was similar to cluster 4 (similarity of 0.289, percentile of 99.096), synergy 3 was identical to cluster 7, and synergy 4 was similar to cluster 1 (similarity of 0.046, percentile of 99.991). In the study group, synergy 1 was similar to cluster 5 (similarity of 0.0525, percentile of 99.956), synergy 2 was similar to cluster 6 (similarity 0.462, percentile of 97.492), synergy 3 was similar to cluster 8 (similarity 0.081, percentile of 99.99), and synergy 4 was similar to cluster 1 (similarity of 0.195, percentile of 98.981). The validity of these similarities, however, should be considered with cautious, especially for clusters that were presented in only few participants, such as clusters 1, 5, 6, and 8 in the study group.

### Validation for the existence of a representative set of synergies

Besides comparing the synergies between groups that account for different movement directions, we also aimed to investigate how the two groups regulate the activation of synergies for different movement directions. Two statistical methods were applied to validate the existence of representative set of synergies: the similarity index and the K-means algorithm. According to the assumption, there exist a representative set of synergies that are activated by different time coefficients during the execution of reaching movements to different directions. The cross validation matrix (Figure 3) confirms that synergies that were extracted from different movement directions can accurately reconstruct EMG's from other movement directions. Specifically, *W*_5_ received the highest mean VAF values of 91.977 ± 1.824 among the other *W* matrices from other targets.

The similarity index was measured for each participant independently, to compute the Euclidian distance between each set of synergies from different directions and the representative set of synergies. Figure 7A illustrates the mean similarity for each group for different movement directions. Figure 7B illustrates the *p*-values of paired *t*-test aimed to analyze the similarity differences between the different targets within groups. For example, it might be asked whether the distance of target 9 to the representative set of synergies is different from the distance of the 1st target from the representative set of synergies. Darker colors indicate significant *p*-values while bright colors indicate non- significant values.

When accounting for the geometric properties of the targets relative to the 5th target at the center of the reaching space, targets 1, 3, 7, and 9 should have the same distance from the representative set, and targets 4, 6, 2, and 8 should have the same distance from the representative set. Therefore, non-significant *p*-values would be expected (i.e., all 7B matrices should be in bright colors). This might explain why target 5's distance from the representative set of synergies was significantly different than the distance of other targets from the representative set of synergies (in both groups).

Figure 7B indicates that the similarities between synergies that were extracted from different movement directions to the representative set of synergies, were different between groups, especially for target 6-9. In the study group, all comparisons between targets were significant. This might imply that post-stroke individuals explore the use of different activation patterns for each different movement direction, specifically to targets that are located in the higher portion of the reaching space. In the control group, on the other hand, participants modulated the activation of the representative synergy in the same manner for different movement direction. Figure 9 illustrates this idea clearly in the control group's participant (9A), and less in the study group's participant (9B) where the activation-coefficients of the four synergies of the representative set of synergies were gradually modified for different directions.

An independent *t*-test was applied to compare the similarity values between groups for each of the movement directions, revealed significant differences between groups for targets 1, 2, 4, and 7 (*p* < 0.05). All comparisons of between-groups synergies revealed greater similarity (lower similarity values) in the control group than in the study group. This suggests that the synergies that were extracted from different movement directions of the control group were closer to the representative set of synergies than the synergies of the study group.

### Discrimination between different movement directions based on the activation coefficient properties of synergies

Using an unsupervised learning approach, the K-means algorithm was applied to cluster the data, based on the activation of synergies. Accordingly, this allowed us to investigate whether the different movement directions can be discriminated based on the activations of four synergies. If it could, it may reinforce the assumption that a representative set of muscle synergies are modulated to control movement for different directions.

Figure 8A demonstrates the resulting indices received by the algorithm for 6 clusters, ordered according to cross-validation matrix in Figure 3. Each row in 8A was ordered in Figure 8B according to the location of the target in space as illustrated in Figure 1B. The mean accuracy of clustering is illustrated in Figure 8C, showing decreased purity values as the number of clusters increased. The mean purity scores were 78.395(7.321)% and 79.382(6.269) in the study and control group, respectively, using nine clusters, 88.024(3.728)% and 86.419(5.672)% for six clusters and 91.851(4.552)% and 91.728(2.732)% for four clusters. High purity scores, with non-significant differences between groups, reinforce the usage of representative set of synergies yielded form the cross validation procedure to express hand-reaching movement for different directions. This especially applied when partitioning the data into four to six clusters. Partitioning the data into higher number of clusters may results in higher incidence of discrimination errors. Figure 8 illustrates an example of six clusters from a single iteration of the K-means of the study group. As Figure 8 illustrates, different movement directions can be accurately discriminated by the K-means algorithm.

### Direction modulation of activation of synergies in the time domain

Another important property for comparing between groups is the time-domain activation of synergies. EMG datasets of each participant from both groups and for each movement direction were decomposed by the representative set of synergies. This was followed by plotting the full activation coefficient matrices to further investigate the activation properties of synergies in the time domain. We additionally calculated the correlations between the chosen features of the full activation coefficient matrices (H) that were factorized by the modified NMF, using *V*_*i*_, *i* ∈ [1, 9] and *W*_5_. Visual inspection of Figure 9A (left) implies there exists high similarities between the activation coefficients of synergies in the time domain between different directions. The correlation matrix on the right side of A confirms this assumption by indicating high correlation values between all pairs of targets. Although subjectively there exists a gradual regulation of the activation coefficient in Figure 9B in the left, the correlation matrix on the right indicates that high correlations exists mainly between targets at the same heights, but less between targets that are in different heights.

## Discussion

Four muscle synergies optimally accounted for variances in the EMG data, which were collected during hand reaching tasks for multiple directions, both in control and in post-stroke individuals. Previous studies also reported that the optimal number of synergies post-stroke were between three and six (Cheung et al., 2012; García-Cossio et al., 2014; Roh et al., 2015; Li et al., 2017). Although it was argued that the number of synergies depends on which and the number of muscles that are monitored (Steele et al., 2013), these studies reported consistent numbers of synergies, independent of the number of muscles that were monitored. Previous studies suggested that increased motor impairments were correlated with a decreased number of synergies, due to merging of healthy synergies (Clark et al., 2010; Cheung et al., 2012; García-Cossio et al., 2014). The findings, however, were noticeable especially in moderate to severely impaired post-stroke individuals (Cheung et al., 2012; Roh et al., 2015) in their chronic stage (Clark et al., 2010; Cheung et al., 2012; Roh et al., 2015). In mildly impaired post-stroke individuals, on the hand, no reduction in the number of synergies was observed compared to healthy individuals (Roh et al., 2012, 2015) or to the less affected hand (Cheung et al., 2012).

Comparisons between synergies of different participants, or between healthy and post-stroke individuals, rely on two assumptions: (A) the synergy control mechanism is valid post-stroke; (B) synergies might be classified as similar or different according to statistical analysis method that discussed earlier. We accordingly applied these comparisons using two different procedures: *First*, comparing between group synergies, which account for different movement directions. This was applied by three methods: (1) direct comparisons between synergies from groups; (2) indirectly, by calculating the similarity of participants' synergies to the representative set of synergies, and comparing these distances between groups, and (3) hierarchical cluster analysis. *Second*, comparing the direction modulation of synergies between groups. This was applied by two methods: (1) the similarity index; (2) the K-means algorithm.

Between-group comparisons for synergies, accounting for different movement directions, revealed significant differences. First, the similarity values, between synergy 2 and the representative set of synergies, in both groups, were high with low percentiles values (Figure 4). These findings were also reflected in the second similarity method, which was indicated that the second and the fourth synergies were differed between groups (Figure 5). Hierarchical cluster analysis was further demonstrated successful portioning the data to seven clusters in the control group, in contrast to poor portioning of the data in the study group. These findings may reflect increased variability in the modulation properties of synergies between post-stroke individuals. The locations of stroke in study group participants were varied among different cortical areas such as the motor cortex in the frontal or parietal lobes, internal capsule, basal ganglia, or thalamus. These findings emphasize the complexity in grouping post-stroke patients as a single group. Accordingly, it is suggested to analyze the synergies in more homogenous group, in terms of the location of the stroke, or to analyze patients' synergies individually.

Other studies found similar synergies post-stroke in mildly impaired individuals, but changes in the structure of synergies in more impaired individuals (Cheung et al., 2012; Roh et al., 2015). Two studies reported that seven clusters partitioned the data, both in healthy and post-stroke individuals (Cheung et al., 2009; Roh et al., 2015). In this study, even though the study group participants sustained mild to moderate motor impairments, the reaching tasks were extremely challenging for most of them. Other studies adjusted the level of exertion necessary to complete the task by post-stroke participants, by reducing the resistance on the hand (Roh et al., 2015), or by adding arm support (Li et al., 2017). Others did not change the tasks between groups (Cheung et al., 2009, 2012). We assume that difficulties in task execution led patients to recruit synergistic muscles, which probably would not have been recruited in less demanding tasks, or if any antigravity support would be have given. Additionally, previous report suggested that isometric contractions did not produce abnormal coupling, indicated by EMG signals, in contrast to dynamic movements (Roh et al., 2012). Accordingly, it might be considered that alteration in the structure of synergies, may reflect the recruitment of additional synergistic muscles, as a compensatory mechanism, which are necessary to complete that task, and not an inherent property of the patient's impairment status.

The modulation of synergies for different movement directions was based on validating the existence of a representative set of synergies. A cross validation procedure between different movement directions was applied to characterize a representative set of synergies, as previously reported (Israely et al., 2017). We further used two statistical methods: the similarity index and the K-means algorithm. These measures were calculated both for validating the representative set of synergies, and also to compare the modulation properties of synergies for different movement directions between groups. The *p*-values in Figure 7B indicate the differences in the distance to the representative set of synergies between different targets. We assume these differences between groups might indicate the deficits in modulating the activation of synergies to different movement directions post-stroke. Figure 9 illustrates this idea clearly in the control group participant (9A), and less in the study group participant (9B) where the activation-coefficients of the four synergies of the representative set of synergies are gradually modified for different directions.

The K-means algorithm revealed equally good classification properties of synergies that were extracted in the cross-validation procedure in both groups, implying that from this point of view, but not as indicated by the cluster analysis, building a representative set of synergies is valid also in post-stroke individuals. It also illustrates that the K-means algorithm may accurately discriminates between movement directions using discrete number of features from the full coefficient matrices post-stroke.

The results of the two indices for modulating synergies between different directions emphasize the difference between synergy analyses and analyses of muscle activation patterns or clinical assessment of movement patterns post-stroke. Post-stroke individuals exhibited stereotyped movement patterns, and decreased capacity to isolate movements of upper extremity segments from each other (Israely and Carmeli, 2016). Respectively, it would be expected to receive higher similarities and higher correlations between synergies from different directions, and decreased ability to classify synergies based on the movement directions. Surprisingly, however, these two measures consistently indicated the opposite. In terms of movement control post-stroke, it might be speculated that in some instances at the spinal level, similar mechanisms are activated to modulate the muscle activity for different directions. Possibly the clinical manifestations of stereotyped pathologic movement patterns, often exhibited post-stroke, are less reflected by the modulation of synergies for different movement direction, but more in the synergy analyses that accounts for the movement directions (As illustrated in Figures 4, 5). Previous studies investigated the modulation of synergies for different direction of isometric force production. Synergy analyzes aimed to discriminate between different movement- directions revealed that both in healthy and in post-stroke individuals synergies were differentially modulated in a task-dependent manner to meet the requirements of the task (Roh et al., 2012). No direct comparisons of modulation properties between groups were reported.

This study is unique in terms of investigating the modulation properties of synergies for different hand reaching directions post-stroke. Post-stroke individuals modulate the activation of synergies differently than healthy individuals, however within patients, a representative set of synergies might be generalized to construct EMG data from different movement directions. Discrete numbers of features, within the full activation coefficient matrices were successfully discriminated between different movement directions in both groups. Several issues should be considered regarding the results of this study. First, the study results may not be generalized for post-stroke individuals with moderate to severe motor impairments. Second, a small number of participants in the study group with large variability in terms of location of stroke, made it difficult to deduce the impact of damage to specific brain area on the modulation properties of synergies. Considering this issue we decided not to construct a representative set of synergies for the study group, but compare synergies of post-stroke individuals to a “healthy” representative set of synergies.

Using muscle synergies to enhance motor performances post lesion to the CNS was previously suggested to be implemented in several ways. Synergies were suggested to be useful for individually tailored rehabilitation protocols (Cheung et al., 2012; Roh et al., 2012). In terms of computational complexity, modulating discrete number of synergies might be easier than controlling large number of degrees of freedom inherent within the nervous system. Accordingly, it was suggested to develop synergy-based biofeedback devices (Safavynia et al., 2011; Roh et al., 2015). Another possibility is to record “healthy” synergies and utilize it to activate damaged extremity of the same person, or to activate prosthetic devices (Roh et al., 2012).

## Conclusions

Two methods for extracting muscle synergies, during hand- reaching tasks for multiple directions, revealed that post-stroke individuals, with mild impairments, differently modulate synergies than healthy individuals. This was demonstrated both when the synergy analysis either accounted or did not account for different movement direction. In contrast to stereotyped movement patterns and decreased ability to dissociate segmental movement post-stroke, two indices unequivocally revealed that post-stroke individual modulate synergies for different movement directions. This demonstrates the differences between analyzing the muscle activation pattern and clinical manifestations of motor impairments and synergy analysis derived by an underlying central control mechanism.

## Author contributions

SI, GL, CM, and EC: Conceived and designed the experiments; SI: Performed the experiments; SI, GL, CM, and EC: Contributed reagents, materials, analysis tools; SI, GL, CM, and EC: Contributed to the writing of the manuscript; GL and EC: supervised the project.

### Conflict of interest statement

The authors declare that the research was conducted in the absence of any commercial or financial relationships that could be construed as a potential conflict of interest.

## Abbreviations

CNS: Central Nervous System
EMG: Electromyography
NMF: Non-negative Matrix Factorization
MVC: Maximum Voluntary Contraction
VAF: Variance Accounted For
MSE: Mean Squares Error.
