# Supplementary material for: Muscle Synergies Control during Hand-Reaching Tasks in Multiple Directions Post-stroke
Source: Front Comput Neurosci. 2018 Feb 23;12:10. doi: 10.3389/fncom.2018.00010 (PMC5829096; doi:10.3389/fncom.2018.00010)
Supplement: Supplementary file 1 [file DataSheet1.DOCX]

**Calculation of percentile of similarity**

Let's assume we have two participants, and we want to calculate the similarity between their synergies and the percentile of similarity.

| participant A | | |  |  | participant B | | |
| --- | --- | --- | --- | --- | --- | --- | --- |
|  | syn1 | syn2 |  |  |  | syn1 | syn2 |
| M1 | a | d |  |  | M1 | a' | d' |
| M2 | b | e |  |  | M2 | b' | e' |
| M3 | c | f |  |  | M3 | c' | f' |

**Stage 1:**

The "real" similarity between participants will be calculated, by the Euclidian distance between the synergy matrices:

**Stage 2:**

Now we want to confirm that the magnitude of similarity that was calculated is not coincidental. Therefore, we will peak randomly ordered synergies (shuffled synergies). Since each synergy has three muscles, so there are 3!=6 possibilities to shuffled each synergy. Then we will compute the similarity between the randomly ordered synergies. Then we will "locate" the values of the "real similarity" within all the values of the shuffled synergies.

|  | Random_ordered_syn1_participant_A | | | |  |
| --- | --- | --- | --- | --- | --- |
| shuffle_A1 | shuffle_A2 | shuffle_A3 | shuffle_A4 | shuffle_A5 | shuffle_A6 |
| a | a | b | b | c | c |
| b | c | a | c | a | b |
| c | d | c | a | b | a |
|  |  |  |  |  |  |
|  |  |  |  |  |  |
|  | Random_ordered_syn1_participant_B | | | |  |
| shuffle_B1 | shuffle_B2 | shuffle_B3 | shuffle_B4 | shuffle_B5 | shuffle_B6 |
| a' | a' | b' | b' | c' | c' |
| b' | c' | a' | c' | a' | b' |
| c' | d' | c' | a' | b' | a' |

| shuffeled_A1 | Vs. | shuffeled_B1 | shuffeled_B2 | shuffeled_B3 | shuffeled_B4 |
| --- | --- | --- | --- | --- | --- |
| shuffeled_A2 | Vs. | shuffeled_B1 | shuffeled_B2 | shuffeled_B3 | shuffeled_B4 |
| shuffeled_A3 | Vs. | shuffeled_B1 | shuffeled_B2 | shuffeled_B3 | shuffeled_B4 |
| shuffeled_A4 | Vs. | shuffeled_B1 | shuffeled_B2 | shuffeled_B3 | shuffeled_B4 |

We will take just 4 shuffled synergies (just synergy1) from the 6 of each of the participants

We will calculate all the possible similarities between the first four randomly shuffled synergies

Since we have two participants, and each have four shuffled synergies, we will receive 16 possible similarity values.

New, let's say that the "real similarity" from stage 1 was **0.25**.

And let's say that the shuffled similarities were (16 possibilities):

0.2, 0.35, 0.4, 0.7, 0.85, 0.1, 0.55, 0.4, 0.33, 0.27, 0.9, 0.72, 0.43, 0.61, 0.29, 0.44

Now we will order the "shuffled similarities" and "locate" the real similarity among the shuffled similarities:

0.1, 0.2, **0.25**, 0.27, 0.29, 0.33, 0.35, 0.4, 0.4, 0.43, 0.44, 0.55, 0.61, 0.7, 0.72, 0.85, 0.9

**Our real similarity value**

Accordingly the percentile of similarity:

$\frac{15}{17}\times100=88.23\%$

Now, we need to decide a threshold value that indicates whether synergies are different or similar (it is advisable that the threshold value will be based on previous studies).

Using this method allowed us to assume that the "real similarity" we received in stage 1 is not a coincidence, and accordingly to decide whether the "inner structure" of the synergies are different or similar.

If the percentile result is above the threshold value so the synergies are assumed to be similar, and if below the threshold value so the synergies are different.
